# Retrograde radical cystectomy and consequent peritoneal cavity reconstruction benefits localized male bladder cancer: results from a cohort study

**DOI:** 10.1186/s12957-015-0561-2

**Published:** 2015-03-31

**Authors:** Xiaojian Qin, Hailiang Zhang, Fangning Wan, Yiping Zhu, Yijun Shen, Bo Dai, Guohai Shi, Yao Zhu, Dingwei Ye

**Affiliations:** Department of Urology, Fudan University Shanghai Cancer Center, No. 270 Dong’ an Road, Shanghai, 200032 China; Department of Oncology, Shanghai Medical College, Fudan University, No. 270 Dong’ an Road, Shanghai, 200032 China

**Keywords:** Bladder cancer, Complication, Peritoneal cavity reconstruction, Prognosis, Retrograde radical cystectomy

## Abstract

**Background:**

Bladder cancer is the second most common genitourinary malignancy. Our study was to introduce a standardized surgical procedure of retrograde radical cystectomy and consequent peritoneal cavity reconstruction in localized male bladder cancer.

**Methods:**

Eighty-four consecutive male patients with localized bladder cancer (clinical stage T2 or lower) underwent surgery in our institute with the proposed procedure between May 2012 and April 2013. Median age was 65 years (range, 35 to 83 years); patient characteristics, surgical parameters, perioperative complications, pathology, and short-term prognosis were analyzed. Median follow-up was 24 months (range, 18 to 30 months).

**Results:**

The complete procedure including urinary diversion took 4.0 h (2.2 to 5.0 h), with a median exposed peritoneal cavity of 45 min (0 to 75 min); the median blood loss was 140 ml (50 to 600 ml), and 2 patients needed transfusion; neurovascular bundles were reserved in 76 cases; the median abdominal and pelvic drainage was 9.0 days (6 to 15 days), the median gastrointestinal recovery was 2.5 days (1 to 12 days), and the median postoperative hospital stay was 13.0 days (10 to 21 days). Four patients had severe surgical complications, and two had mild to moderate ileus, with recovery in 1 and 2 weeks with supportive treatment. No perioperative deaths or postoperative recurrence were reported.

**Conclusions:**

The surgical procedure in male localized bladder cancer described in the present study provided surgical facilities, with limited abdominal organ disturbance and satisfactory tumor control. The procedure was associated with good gastrointestinal recovery, few postoperative complications, and a short hospital stay.

## Background

Bladder cancer is the second most common genitourinary malignancy [1]. Radical cystectomy (RC) and pelvic lymph node dissection (PLND) are the standard treatments for muscle invasive bladder cancer (MIBC) and a valid option for selected patients with high-grade non-muscle invasive bladder cancer (NMIBC) [2-4]. Improvements in surgical techniques and modern perioperative care have substantially decreased the rate of perioperative complications and lowered the operative mortality rate [5]. However, this procedure remains complication-prone and is associated with significant perioperative and long-term morbidity ranging from 19% to 64% according to different series [6].

The most commonly used surgical technique is the descending transperitoneal approach. In this procedure, the peritoneum covering the bladder is incised together with the bladder. Early complications, which occur in 20% to 58% of patients after RC and PLND [7-9], consist mostly of gastrointestinal motility disorders, which occur in almost one third of patients [10]. An extraperitoneal procedure with extraperitonealization of the ileal bladder was introduced to reduce morbidity. This modified procedure significantly improved postoperative recovery of bowel function [11-16].

However, the indications for extraperitoneal cystectomy have not been fully defined. The peritoneum is one of the most frequent sites of metastases in bladder cancer [17,18]. Many patients who undergo extraperitoneal cystectomy are lymph node positive or have non-organ-confined disease [11,13], and the possible presence of residual tumor cells on the preserved peritoneum is an issue of concern. A previous study from our group showed that tumor stage and lymph node status are independent predictors of peritoneal involvement. Therefore, extraperitoneal cystectomy should be performed with caution and only in patients with a high likelihood of stage pT1 and pT2. In patients with stages cT2 to 4, positive lymph nodes, or non-urothelial histologies, the peritoneum covering the bladder should not be preserved [19].

Previous studies on extraperitoneal cystectomy indicated the importance of preserving as much peritoneum as possible to reduce postoperative complications and to improve gastrointestinal recovery. In the present study, we expand this concept by introducing peritoneal cavity reconstruction (PCR) after retrograde radical cystectomy (RRC) for tumor control and functional preservation. The aim of the present study was to introduce a standardized surgical procedure of RRC and consequent PCR for the treatment of localized bladder cancer in men.

## Methods

Eighty-four consecutive male patients with localized bladder cancer (clinical stage T2 or lower) underwent RRC and PCR in our institute between May 2012 and April 2013. Of these patients, 19 were clinical stage T1 and 65 were clinical stage T2. Median age was 65 years (range, 35 to 83 years). All patients were staged preoperatively with spiral pelvic computed tomography (CT) and abdominal ultrasonography. Transurethral resection of bladder tumors extending to the deep muscle layer was performed in patients whose clinical stage was uncertain by CT. Abdomen magnetic resonance imaging or CT was used to rule out metastases to abdominal organs and retroperitoneal LNs. None of the patients underwent neoadjuvant systemic chemotherapy. The median preoperative body mass index was 22.5 kg/m^2^ (16.9 to 27.7 kg/m^2^), and the median chronic comorbid diseases (comorbidities) score according to the Charlson comorbidity index was 6 (2 to 9) [20]. The local ethics committee of Fudan University Shanghai Cancer Center approved the study, and all patients gave their informed consent.

**Figure 1:**
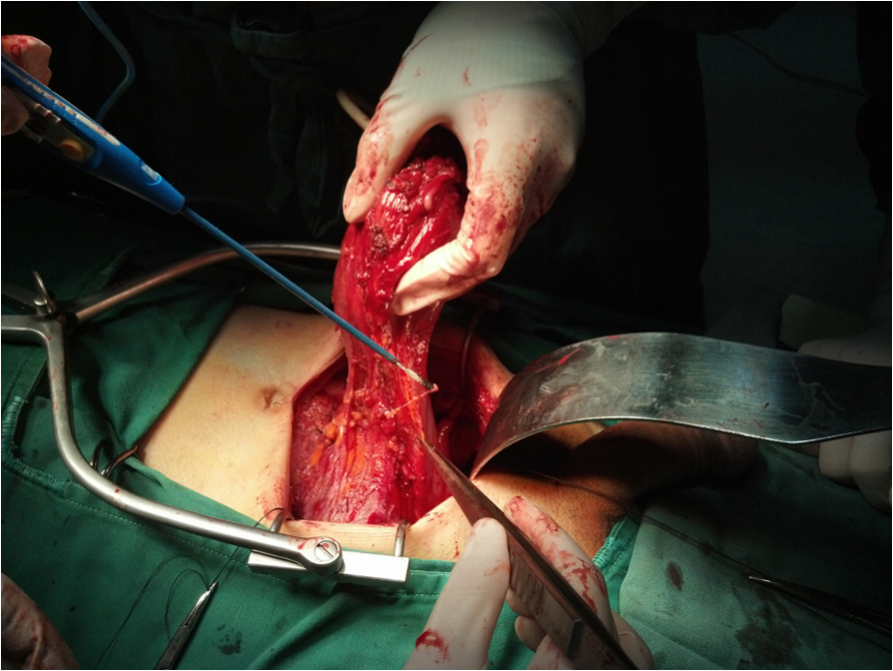
**The dissected prostate and bladder are pulled up, with only peritoneum at the level of the bladder dome attached to the surrounding peritoneal flaps.**

**Figure 2:**
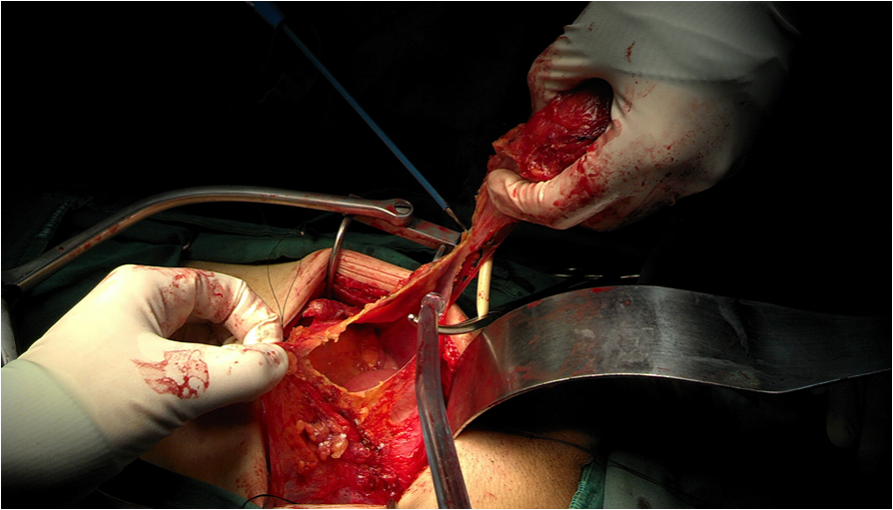
**Preservation of the maximum possible amount of peritoneum.**

**Figure 3:**
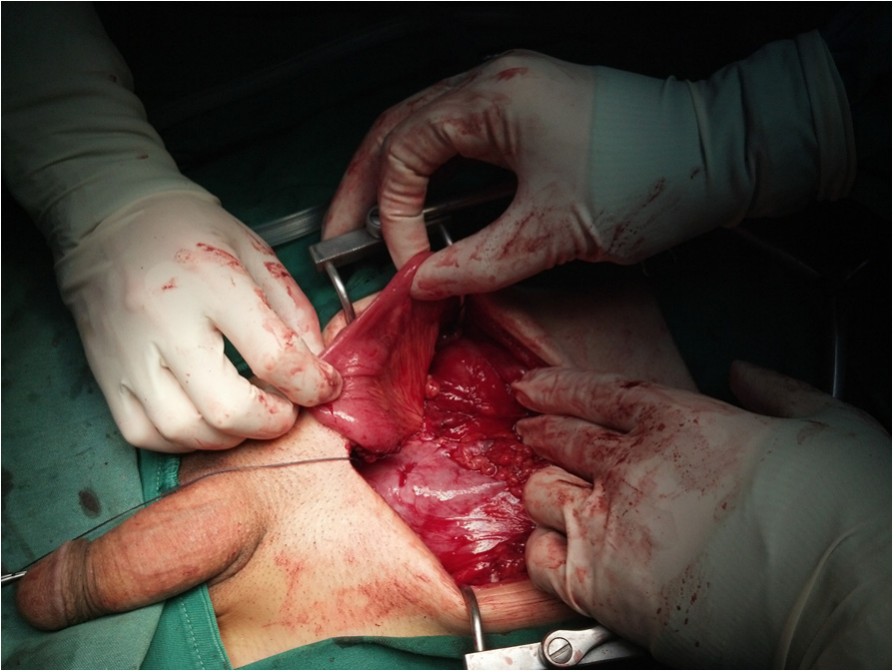
**Reconstruction of the peritoneal cavity.**

Extraperitoneal surgery was performed as described by Kulkarni *et al*. [15] with certain modifications. Briefly, a midline infraumbilical incision was performed regardless of whether a conduit or neobladder was reconstructed. The space of Retzius was entered inferiorly and a standard bilateral PLND was performed before the cystectomy, including the nodes along the distal half of the common iliac vessels together with its bifurcation. Unlike the procedure described by Kulkarni *et al*., the ureters close to the ureterovesical junction were identified and divided, and ureteral frozen section analysis was performed until negative ureteral margins were achieved bilaterally. During the frozen section analyses, retrograde retropubic dissection of the prostate and the bladder was performed following the procedure described by Kulkarni *et al*. The dissected prostate and bladder were pulled up, with only peritoneum at the level of the bladder dome attached to the surrounding peritoneal flaps (Figure 1). In patients with clinical NMIBC, the peritoneum was preserved as much as possible, and in those with clinical MIBC, the peritoneum was completely removed together with the bladder. This minimized peritoneal defects, and the remaining peritoneum could be expanded sharply to the right side for neobladder or ileal conduit reconstruction (Figure 2). Before urinary tract reconstruction with the neobladder or ileal conduit, the peritoneal cavity was reconstructed after isolation of an ileal loop, reestablishment of bowel continuity cephalad to the ileal loop, and closure of the mesenteric window (Figure 3).

**Figure 4:**
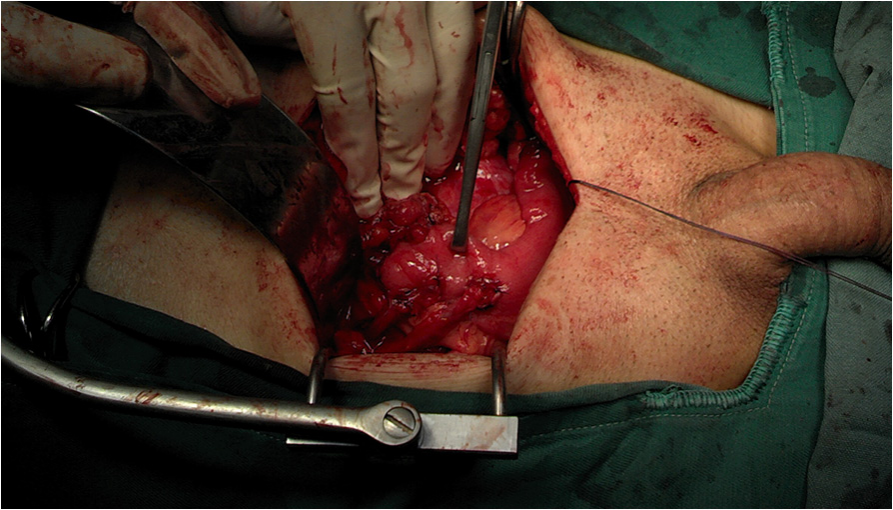
**Cutaneous ureterostomy and preparation of the ileal loop, neobladder or ileal conduit reconstruction, and urinary reconstruction are performed extraperitoneally.**

Cutaneous ureterostomy, ileal neobladder, or ileal conduit was applied as urinary diversion in the present cohort. Cutaneous ureterostomies were applied in patients who were too old or with severe comorbidities and could not tolerate long operation time. Since ileal conduits are relatively easy and quick to create, minimising the rate of postoperative complications, ileal conduits remain the ‘gold standard’ for incontinent diversion, as was in the present cohort. Orthotopic ileal neobladders were usually applied in those patients with reliable active postoperative participations to ensure proper maintenance of the reservoir. All procedures of urinary diversions were carried out without compromising oncologic control, after discussion with the patients and written contracts were obtained. Among the 84 patients, 51 underwent ileal conduit, 29 underwent ileal neobladder, and 4 underwent cutaneous ureterostomy. In patients undergoing cutaneous ureterostomy, the peritoneal cavity is closed shortly after the procedure or may not need to be opened if the cancer is clinically superficial. Cutaneous ureterostomy, as well as the preparation of the ileal loop, the neobladder or ileal conduit reconstruction, and urinary tract reconstruction can be performed extraperitoneally (Figure 4). Bilateral pelvic or peritoneal cavity drainage tubes were removed when the output remained at <30 ml.

Surgical parameters, perioperative complications, pathology, and short-term prognosis were documented. Surgical parameters included the number of lymph nodes dissected in PLND, neurovascular bundle preservation, operation time, duration of abdominal organ exposure (time interval between the peritoneal incision and the closure of the peritoneal cavity), blood loss, transfusion rate, and abdominal drainage time. Early complications were recorded within 90 days of surgery or during patient hospitalization. In accordance with a recent review of various complications following RC [21], a checklist was drafted to identify complications from medical records. Complications were defined by clinical and laboratory examinations with/without radiological evaluation. The severity of complications was evaluated using the Clavien-Dindo classification (CDC) [22]. Any complication of grade 3 or higher was defined as a severe complication. Postoperative hospital stay and perioperative ileus were monitored carefully. All the resected samples were subjected to pathologic analyses. Patients remained in the hospital for an additional 2 to 3 days to monitor for potential complications after removal of drainage tubes, and all patients were followed-up at the outpatient department after discharge.

The median follow-up period was 24 months (range, 18 to 30 months). None of these patients received adjuvant chemotherapy, and all of them were followed up every 3 months after surgery. Physical examination and imaging (abdominal ultrasound, thoracic radiography, CT of the pelvis) were undertaken to detect local recurrences and distant metastases.

## Results and discussion

Surgical parameters were listed in Table 1. In two patients who underwent cutaneous ureterostomy, opening of the peritoneal cavity was not necessary and the peritoneum attached to the bladder dome remained intact. Surgical complications were CDC grade 2 in 27 patients and grade 3 or above in 4 patients; there was no rectal injury during the operations. Two patients developed mild to moderate postoperative ileus and recovered within 1 and 2 weeks with supportive treatments. No perioperative deaths were reported. Perioperative complications are listed in Table 2.

**Table 1:** **Surgical parameters of 84 male patients who underwent RRC and consequent PCR**

**Lymph nodes removed (number)**	**8 to 16 (median, 12)**
Duration of complete procedure (hours)	2.2 to 5.0 (median, 4.0)
Exposure of peritoneal cavity (minutes)	0 to 75 (median, 45)
Blood loss (ml)	50 to 600 (median, 140)
Transfusion (number)	2
Neurovascular bundles preserved (number, unilateral, or bilateral)	76
Abdominal and pelvic drainage (days)	6 to 15 (median, 9.0)
Gastrointestinal recovery (days)	1 to 12 (median, 2.5)
Postoperative stay (days)	10 to 21 (median, 13.0)

PCR, peritoneal cavity reconstruction; RRC, retrograde radical cystectomy.

**Table 2:** **Detailed perioperative complication of 84 male patients who underwent RRC and consequent PCR**

**Category**	**Complication**	**Total (** ***n*** **)**
Gastrointestinal	Ileus^a^	2
	Constipation^b^	1
	Gastrointestinal bleeding	1
	Bowel leak	0
	Clostridium difficile colitis	0
	Gastric ulcer	1
Infectious	FUO	1
	UTI	12
	Sepsis	1
	Pyelonephritis	1
	Gastroenteritis	0
	Cholecystitis	0
	Pelvic abscess	2
Wound	Wound dehiscence	1
	Surgical site infection	1
Genitourinary	Renal failure	0
	Hydronephrosis	2
	Urinary leak	1
	Necrosis of ileal conduit	0
	Parastomal hernia	0
	Testitis	1
Cardiac	Arrhythmia	1
	Myocardial infarction	1
	Acute heart failure	1
Pulmonary	Respiratory distress	0
	Pneumonia	1
Bleeding	Anemia requiring transfusion	2
	Postoperative bleed other than GI	0
Thromboembolic	Deep venous thrombosis	1
	Pulmonary embolism	0
Neurological	Peripheral neuropathy	1
	Delirium/agitation	0
Surgical	Vascular injury	1
	Anastomotic bowel leak	0
	Rectum injury	0
Miscellaneous	Lymphatic leak	11

*n*, the total number of patients within that category; FUO, fever of unknown origin; UTI, urinary tract infection; GI, gastrointestinal; PCR: peritoneal cavity reconstruction; RRC: retrograde radical cystectomy. ^a^Ileus is defined as postoperative nausea or vomiting associated with abdominal distension confirmed by radiological examination. ^b^Constipation is defined as inability to have a bowel movement by postoperative day 5 with no signs of ileus or small bowel obstruction. Infectious complications were diagnosed by positive culture. Prolonged lymphatic leak is defined as more than 100-ml drainage output for 2 days starting from postoperative day 3.

The diagnosis of urothelial cell carcinoma was confirmed by postoperative pathology in the 84 patients. Of 19 patients with cT1 disease, 16 were stage pT1 and 3 were upstaged to pT2 disease. Among the 65 patients with cT2 disease, 51 remained in pathologic stage pT2 and 14 were upgraded to pT3a. Pelvic lymph node metastases were concurrently found in five patients with MIBC. No cases of recurrence were detected during the follow-up period.

The most common surgical technique in patients undergoing RC is the descending transperitoneal approach. However, this procedure is associated with several postoperative complications. The peritoneal cavity is entered early in the procedure, leading to unnecessary bowel exposure to the atmosphere. Mobilization of the bowel often disturbs the operative field during cystectomy. Packing the bowels into the upper abdomen may cause mechanical damage. The increasing depth of dissection is associated with a risk of accidental entry into the bladder or rectum and poor exposure of the prostatic apex and rectal surface, leading to tumor spillage or contamination, as well as difficulty preserving the neurovascular bundle and striated sphincter. Large defects in the peritoneum contribute to the lack of a barrier protecting the abdominal organs [7-9,23].

In the present study, we introduce a modified surgical technique for extraperitoneal RC and a novel concept of subsequent PCR, in which the peritoneal cavity is rebuilt after radical dissection of the bladder and before urinary reconstruction using residual peritoneum. This procedure is made feasible by preserving as much peritoneum as possible in a retrograde extraperitoneal manner. In the extraperitoneal approach, opening of the peritoneum is minimized during RC and PLND, and the peritoneal cavity is reclosed before urinary reconstruction. The application of the extraperitoneal approach to the treatment of bladder cancer has been discussed extensively, and the retrograde extraperitoneal approach requires a thorough knowledge of the anatomy of the extraperitoneal space and methodical dissection, making it inherently superior to the conventional method [11-16]. In the present cohort, the procedure was performed with short operating times, limited exposure of abdominal organs, limited blood loss, low transfusion rates, and high neurovascular bundle preservation rates. The modified procedure described in the present study was beneficial in terms of the surgical technique and limited abdominal organ disturbance with respect to tumor control. Moreover, the notion of subsequent PCR provides a rationale for the preservation of the peritoneum during extraperitoneal procedures.

The peritoneum is a natural barrier that isolates the small bowel and pelvic organs. The extraperitoneal technique provides an undisturbed operative field during cystectomy and obviates any need to pack the bowels into the upper abdomen. The duration of bowel exposure to the atmosphere is also decreased. As reported by Kulkarni *et al*. [15], the preservation of large peritoneal flaps is beneficial during repeat peritonealization to isolate the bowel anastomoses, which is created after harvesting the bowel loop for neobladder or conduit reconstruction. This technique protects the urinary anastomoses from the septic complications of bowel anastomosis.

In the present study, most patients showed no or mild complications and none required aggressive treatments. CDC grade 2 complications requiring medical intervention were mainly lower urinary tract infections, which were controlled by adjustment of antibiotics and monitoring by urine cultures. Mild to moderate postoperative ileus occurred in two patients, who showed recovery within 1 and 2 weeks with supportive treatment. CDC grade 3 or higher complications that were life-threatening or required surgical intervention were rare, and there were no perioperative deaths. Two patients developed pelvic infections requiring redrainage. Since the abdominal organs were isolated from the pelvic space after abdominal reconstruction, infections were confined to the pelvis and did not lead to serious complications in the abdominal cavity. Patients were treated conservatively, and drainage tubes were removed when the output remained below 30 ml. In addition, patients were observed for an additional 2 to 3 days to monitor for potential complications after the removal of drainage tubes. Despite differences in patient management protocols among institutions, we found that the present procedure shortened hospital stay and reduced the risk of serious comorbidities during follow-up.

Our retrograde extraperitoneal surgical approach seems to mainly affect the extent of PLND, for which there is no consensus among clinicians and academic professionals. Although extended lymphadenectomy has been shown to improve survival in patients with both lymph node-negative and limited lymph node metastatic disease [24], extensive removal of lymph nodes is associated with certain complications, such as lymphatic fistula, bleeding, and lower extremity lymphedema. In the present cohort, we generally applied the ‘standard’ dissection, which proceeds from the medial section to the genitofemoral nerve as the lateral limit of node dissection. The entire external iliac artery and vein are dissected up to 2 cm above the bifurcation of the common iliac artery. Previous studies suggest that this extent of PLND covers more than 80% of lymph nodes in the lymphatic drainage field [25-27]. The median number of lymph nodes dissected in the present cohort was 12, and the positive rate was 6% (5/84). None of the patients received adjuvant chemotherapy, and all of them were followed-up every 3 months after surgery, with no cases of recurrence. Although longer follow-up is needed to draw a final conclusion, the preliminary results indicate that our introduced procedure yielded a reliable oncological control.

In our previous study analyzing a patient population that underwent transperitoneal RC, the peritoneum covering the bladder was detached after removal of the bladder. Any suspicious macroscopic abnormalities of the peritoneum were sampled and examined. A 6- to 8-point random biopsy of the peritoneum was performed in patients without gross macroscopic abnormalities. In that study, 5 of 34 patients with pT3 (14.7%) and 3 of 13 patients with pT4 (23.1%) had positive biopsies; among patients with positive lymph nodes, 5 of 34 patients (14.7%) had positive biopsies [19]. In addition, 15% to 43% of patients staged cT1 were upstaged to pT2 at pathological examination of the radical cystectomy specimen. The results were even worse for patients with cT2 disease, among which 35% to 77% were upstaged [28-30]. In the present study, 3 of 19 cT1 patients were upstaged to pT2 disease, and 14 of 65 cT2 cases were upstaged to pT3a. Pelvic lymph node metastases were concurrently found in five patients with MIBC. To reduce the risk of residual tumor in the preserved peritoneum, we selected the candidates very carefully, and only recommended those with localized bladder cancer (clinical stage T2 or lower) in the present cohort. Furthermore, enough peritoneum covering the bladder was resected in an en bloc manner together with the bladder.

The present study had several limitations. The analysis was limited to men with clinically localized bladder cancer, which may restrict its immediate application in more advanced diseases. In addition, because of anatomical differences between males and females, whether this introduced procedure could be reproduced in females warrants further discussion. As a cohort study, the present analysis has limited power to conclude that the introduced procedure is superior to others with respect to oncological control or functional preservation. A randomized trial is necessary to address these concerns. Moreover, the present cohort did not document some long-term follow-up outcome evaluation like renal function and neobladder function or evaluate the long-term quality of life. Nevertheless, the modified procedure of retrograde extraperitoneal RC showed beneficial results, suggesting that it could reduce tumor residue during extraperitoneal procedures. Furthermore, the introduction of PCR after RRC may stimulate further investigation into this technique. As a single-center cohort report, further prospective studies in other centers are needed for validation.

## Conclusions

The surgical procedure in male localized bladder cancer described in the present study provided surgical facilities, with limited abdominal organ disturbance and satisfactory tumor control. The procedure was associated with good gastrointestinal recovery, few postoperative complications, and a short hospital stay. Further investigation into the proposed procedure is thus warranted.

## Abbreviations

CDC: Clavien-Dindo classification
CT: computed tomography
MIBC: muscle invasive bladder cancer
NMIBC: non-muscle invasive bladder cancer
PCR: peritoneal cavity reconstruction
PLND: pelvic lymph node dissection
RC: radical cystectomy
RRC: retrograde radical cystectomy
